# Exploring the effects of habituation and scent in first-person 360-degree videos on consumption behavior

**DOI:** 10.1038/s41598-023-35669-5

**Published:** 2023-05-23

**Authors:** Benjamin J. Li, Hui Min Lee

**Affiliations:** Wee Kim Wee School of Communication and Information, Nanyang Technological University, WKWSCI Building #03-17, 31 Nanyang Link, Singapore, 637718 Singapore

**Keywords:** Human behaviour, Nutrition

## Abstract

Although immersive virtual environments can influence food-related thoughts, emotions and behavior, the influence of repeated exposure to food cues in such environments has rarely been explored. This study seeks to understand if habituation, a decrease in one’s physiological and behavioral response that results from repeated simulation, can take place while repeatedly watching 360-degrees of food being consumed. The influence of scent as an olfactory cue is further explored, based on past research on embodied cognition. In Study One (*n* = 42), participants who viewed 30 repetitions of someone eating an M&M ate significantly fewer M&Ms than those who viewed three repetitions. Study Two (*n* = 114) used a 2 (behavior: eating M&M/inserting a coin) × 2 (repetitions: 3/30) between-subjects experiment to confirm that results from Study One were due to habituation of the consumption video, finding that there were only significant differences between repetitions in the M&M condition. Finally, Study Three (*n* = 161) comprised a 2 (repetition: 3/30) × 2 (scent: present/absent) between-subjects experiment. Participants in the 30-repetition condition and those in the scent-present condition ate significantly fewer M&Ms respectively, but no interaction effects were found. The theoretical and practical implications of these findings are discussed.

## Introduction

Amongst its many uses, virtual reality (VR) exceedingly demonstrates potential as a suitable medium to study consumption behavior. Studies have shown that engaging in VR experiences can influence food-related thoughts, emotions, and behavior^1–3^. The virtual environment immerses individuals and creates a lifelike environment where individuals can be exposed to food cues or related scenarios, inducing responses such as craving and salivation^1,4^. In fact, VR environments may even have stronger effects on certain food-related responses than real-life exposure^5^.

However, research shows that repeated exposure, or even repeated imagining, of food cues may have reverse effects^6,7^. For example, Morewedge et al.^6^ found that repeatedly imagining consuming an M&M decreased actual consumption of M&Ms. This is a phenomenon that is commonly explained by *habituation*, which refers to a decrease in one’s physiological and behavioral response that results from repeated simulation^8^. Habituated individuals thus become less motivated to respond to the food cue, showing less desire to obtain or consume the food^6,9^. As a result, cue exposure therapy, where obese or binge-eating individuals are repeatedly exposed to food cues in the virtual environment, is a common form of therapy^10^. Repeated exposure reduces the mental association between the food cue and one’s over-eating response, attenuating individuals’ food cravings and decreasing consumption.

Despite various studies examining the effects of repeated exposure to food cues or repeated imagining of food^6,11^, few studies have explored repeated exposure within a virtual environment. Getting individuals to imagine large repetitions of an action may be cognitively taxing, and it may also not be practically feasible to repeatedly expose people to real-life settings where problematic eating behaviors commonly take place^10^. The virtual environment hence presents an alternative medium to influence consumption behavior.

In particular, 360-degree videos, also known as immersive videos, are often considered a type of VR technology and content^12,13^ and may influence viewers in ways that are similar to immersive VR experiences^12^. As 360-degree videos are often easier to access and create than interactive VR experiences, there is practical value in understanding if such videos may equally influence consumption behavior and whether they may be used in food-related therapy or treatments.

Epstein et al.^14^ also suggest that habituation may occur across modalities. The virtual environment, with its ability fully immerse the user through various senses, may therefore be an appropriate medium to test the effects of habituation across modalities. In fact, studies on habituation have shown that olfactory cues may influence habituation^15^, while VR consumption studies have found effects of olfactory cues as well^1,16^. As humans experience the world through a multitude of senses, could the addition of olfactory cues in a virtual environment better approximate a real-world setting, having effects on habituation to food cues and resultant consumption behavior?

This paper therefore explores the effects of habituation in 360-degree videos. We test the effects of repeated exposure to food cues on consumption behavior (Study One and Two) and explore how olfactory cues may further influence this behavior (Study Three).

### Habituation to food cues

Habituation refers to a decrease in one’s physiological and behavioral response that results from repeated simulation^8^. Put simply, while we may initially get distracted by the sound of a phone ringing, we become more accustomed and pay less attention to it when it has been left ringing for a long time. This phenomenon occurs because information stored in one’s short-term memory may fail to be surprising to the individual over repeated exposure^17^. The individual therefore becomes less motivated to process the stimuli, responding to it less over time^14^.

In the case of consumption behavior, habituated individuals become less motivated to respond to the food cue, showing less desire to obtain or consume the food^6,9,18^. Studies have therefore shown that individuals habituate to food cues through decreased salivary responses^19^, decreased motivational response to obtain food^20^, and most prominently, decreased consumption behavior^20,21^.

Although most habituation studies repeatedly expose individuals to food cues, a study by Morewedge et al.^6^ found that habituation can also occur through vivid imagination of *consuming* food. In the study, participants were tasked to imagine themselves repeatedly consuming M&Ms. Results showed that merely imagining consuming an M&M 30 times led to a significant decrease in consumption of M&Ms, as compared to imagining consuming it only once. In a similar study, Missbach et al.^11^ found that compared to those who imagined inserting a coin into a laundry machine, participants who repeatedly imagined consuming gummy bears showed decreased consumption of gummy bears. Other studies have supported these findings, demonstrating habituation effects across the repeated imagination of consuming other types of food such as walnuts and even alcohol^21,22^.

Notably, habituation that occurs via repeated imagining may also be associated with sensory-specific satiety, which refers specifically to the decrease in hedonic liking for a food upon repeated consumption or exposure^23^. A key principle underlying habituation is that a presented stimuli undergoes a change of state from the initial exposure to the food (A1), to its subsequent exposures (A2), and finally a state of habituation (I). The A1 state involves active processing of the sensory information related to the food, activating the memory node to store information regarding the food. The A2 state then makes use of more peripheral processing, while the I state occurs when the brain has already formed an association with the food item and therefore becomes less motivated to respond to it.

Sensory-specific satiety may therefore be a “special case of habituation theory”^14^, whereby the repeated imagining of consuming a specific food initially triggers a highly active state and activates the autonomous nervous system akin to how we prepare to eat, but this weakens to a more peripheral form of processing over repeated exposure, decreasing hedonic liking for the food at the same time. As this study is ultimately concerned with how repeated exposure to consuming a food in the virtual environment can influence actual consumption behavior, we view sensory-specific satiety as a type of habituation, especially where repeated exposure or imagination of consuming a food is concerned.

### Virtual environments, food cues, and consumption behavior

An increasing number of studies have explored the effects of virtual environments on consumption behavior, showing how such environments can influence food craving and even actual consumption behavior^1–3,24^. For example, van der Waal et al.^3^ found that both VR and real-life chocolate cues led to stronger cravings, while Gorini et al.^5^ found similar effects amongst those with eating disorders. In fact, a systematic review of consumption behavior in VR settings concluded that VR can create an authentic environment that induces emotional reactions related to food consumption in individuals^25^. Exposure to food cues in the virtual environment can therefore increase craving, or desire, for food.

From a technical standpoint, virtual environments are immersive media that often display a high level of sensory fidelity, offering users visual and auditory cues similar to those experienced in the real world^26^. Virtual environments that fully surround and immerse the user may thus create the psychological sensation that one is in the virtual environment, also known as a sense of presence^27^. Because exposure to different types of cues in a VR environment can strongly replicate the complex natural environment and lead to a sense of “being there”, people often respond to VR as they would in real life^24^. Individuals who are shown a food in VR may therefore respond as if they were shown a real food.

However, the effects of *repeated exposure* to consuming food in virtual environments have not been fully explored. Since exposure to food cues in virtual environments may have similar effects on individuals’ craving as in real life, we believe that repeated exposure to consuming food would also induce habituation in participants. Most VR studies on reducing consumption behavior or treating eating disorders only focus on immersing individuals in everyday environments, such as a kitchen, dining room or cafeteria^28,29^, while others have used interactive VR experiences, allowing individuals to interact with virtual food cues^24^. It thus remains unclear if repeated exposure in a 360-degree video may similarly result in decreased consumption.

Compared to merely repeatedly imagining consumption, which relies on one’s cognitive resources, the virtual environment may also present a more robust medium to induce habituation effects through repeated exposure to consuming food^21^. This is supported by past studies on habituation, which found that habituation from repeated imagining may only occur in those with self-regulatory resources^11^. In contrast, being immersed in 360-degree videos may easily facilitate a sense of presence, where individuals then respond to the virtual stimuli as they would to real-life stimuli. The virtual environment therefore better simulates real-life food intake for habituation effects to occur. Watching 360-degree videos of consuming a food can thus be similar to repeated imagining of a consuming a food, being an equally powerful immersive experience that induce habituation effects and decrease consumption behavior, similar to repeated imagining of a stimulus^12,30^.

### Effects of olfactory cues

Humans experience the world through a multitude of senses, and this particularly applies to food consumption. Eating a plate of fried chicken involves not only visual cues of it, but also hearing its crunch as you bite into it, and smelling the fried oil that permeates the environment. In fact, of the five senses, olfactory cues have been estimated to contribute as much as 80 to 90% of flavor perception^31,32^. Studies have therefore shown that exposure to olfactory food cues may have effects on responses such as the amount of food consumed and taste perception^1,7,33,34^.

However, the effects of olfactory cues on food-related responses appear somewhat varied. Some studies have found that olfactory cues can increase individuals’ craving and food responses, for instance, Firmin et al.^35^ found that inhaling a sweet scent increased individuals’ craving for chocolate, while another study found that smelling palatable stimuli such as cakes and pastries increased consumption among certain individuals^36^. This is due to mental imagery of the palatable food item, and is further supported by a finding that imagining an odor can enhance food-related responses when accompanied by vivid visual imagination of the stimuli^37^.

However, other studies demonstrate that olfactory cues can also reduce consumption. Prolonged exposure to a scent may induce a satiation effects—Rolls and Rolls^38^ found that smelling a food for five minutes decreased individuals’ rating of how pleasant the food was, while Biswas and Szocs^39^ concluded that extended exposure to scents of indulgent food such as cookies can reduce preference these unhealthy foods. In fact, when individuals smelled an odor of a food for the duration it took to consume that food, activity in their orbitofrontal cortex, an area of the brain involved in processing smells, decreased^40^. This supports other studies finding that short exposure (30 min) to chocolate odor increased chocolate craving, but long exposure (60 min) showed decreased craving^41^.

The interactive effect of visual and olfactory cues is also a topic of discussion^33,34,37^. While some studies show that olfactory cues can enhance the effect of visual cues on craving towards the food item^34^, a model by Kavanagh et al.^42^ suggests that introducing additional imagery associated to one’s craving, such as introducing olfactory cues to imagining visual food cues, may compete for limited cognitive resources and thus decrease food craving and consumption. This notion is supported in a study by Kemps and Tiggemann^43^ that found that exposure to a neutral, unfamiliar odor alongside pictures of foods that contained chocolate reduced the desire for it. Overall, the mixed findings regarding the effects of olfactory cues suggest that the overall environment, the type of olfactory cue, and the duration of exposure to the cues, are important factors determining craving and consumption behavior.

In the context of a VR environment, the effects of olfactory cues can be further explained by the experience of embodied cognition. According to Barsalou^44^, in the absence of a real experience, one retrieves the perceptual symbols of these experiences that were stored in their memory, and their mental simulations lead to such an activation of how they would respond as if they were in real life. For example, individuals who read words such as “garlic” or “cinnamon” demonstrated an activation of their primary olfactory cortex^45^. When individuals are presented with the scent of a desirable food in VR, this may contribute to a mental simulation of eating the food, inducing satiation effects, and decreasing consumption. This was evidenced in a study that found that participants exposed to olfactory cues while interacting with a donut in VR consumed fewer donuts as compared to their counterparts^1^. Theoretically, the virtual environment’s ability to fully immerse the user may therefore create a realistic experience akin to consuming the food in real life and decrease actual consumption behavior.

Xu et al.^25^ further suggest that a wider range of modalities within a VR system, such as providing users with visual, auditory and olfactory cues, as compared to only visual and auditory cues, can increase immersion of the system. The immersive virtual environment may enhance individuals’ feelings of presence, allowing them to respond more accurately to the food cues presented. This has been supported in other studies that have found that exposure to olfactory cues in a VR setting increased sense of presence^16,46^, meaning that individuals perceived the virtual environment to be similar to real life and would therefore respond like they would in real life. Individuals can therefore experience habituation in the form of embodied cognition from prolonged exposure to olfactory cues in the virtual environment. Thus, despite the mixed findings regarding olfactory cues, this study posits that prolonged exposure to olfactory cues, arising from the repeated exposure to visual cues of a food, in a virtual environment would reduce consumption.

## Methods and results

To test our propositions, three studies were conducted. Study One and Two examined the effects of repeated exposure to food cues on consumption behavior, while Study Three explored how olfactory cues may further influence this behavior. The three studies conducted and reported here were approved by the Institutional Review Board of Nanyang Technological University, and were conducted in accordance with institutional ethical guidelines and regulations. Participants in all studies comprised college students who were approached through the university’s electronic mailing lists. As they were required to consume food, students who reported as diabetic or allergic to chocolate were not allowed to sign up. All participants provided informed consent at the start of the study and received monetary compensation for their time.

### Study One

Study One examined if repeatedly watching a 360-degree video of M&Ms being consumed would lead to decreased consumption behavior. Based on the effect size of 0.48 in Morewedge et al.^6^ study, power analysis showed a target sample size of 45. As such, we recruited a total of 48 participants. However, six students dropped out before the start of the study, leading to a final sample of 42 participants. Participants were equally distributed across the three conditions (*n* = 14), consisted of 23 males (54.7%) and their ages (*M* = 22.4, *SD* = 1.57) ranged from 21 to 26.

#### Method

The study comprised three conditions. In the control condition, participants watched a coin being inserted into a laundry machine 33 times. This was previously demonstrated as being an action that involves motor skills similar to those exhibited in the eating condition, yet different enough in context from consumption behavior^6^. In the three-repetition condition, participants watched a coin being inserted into a laundry machine 30 times, followed by three repetitions of watching an M&M being consumed from their perspective. In the 30-repetition condition, participants watched a coin being inserted into a laundry machine three times, followed by 30 repetitions of watching an M&M being consumed. The inclusion of the coin being inserted into the washing machine in both conditions ensured that all participants spent the same amount of time watching the video in their respective conditions.

**Figure 1:**
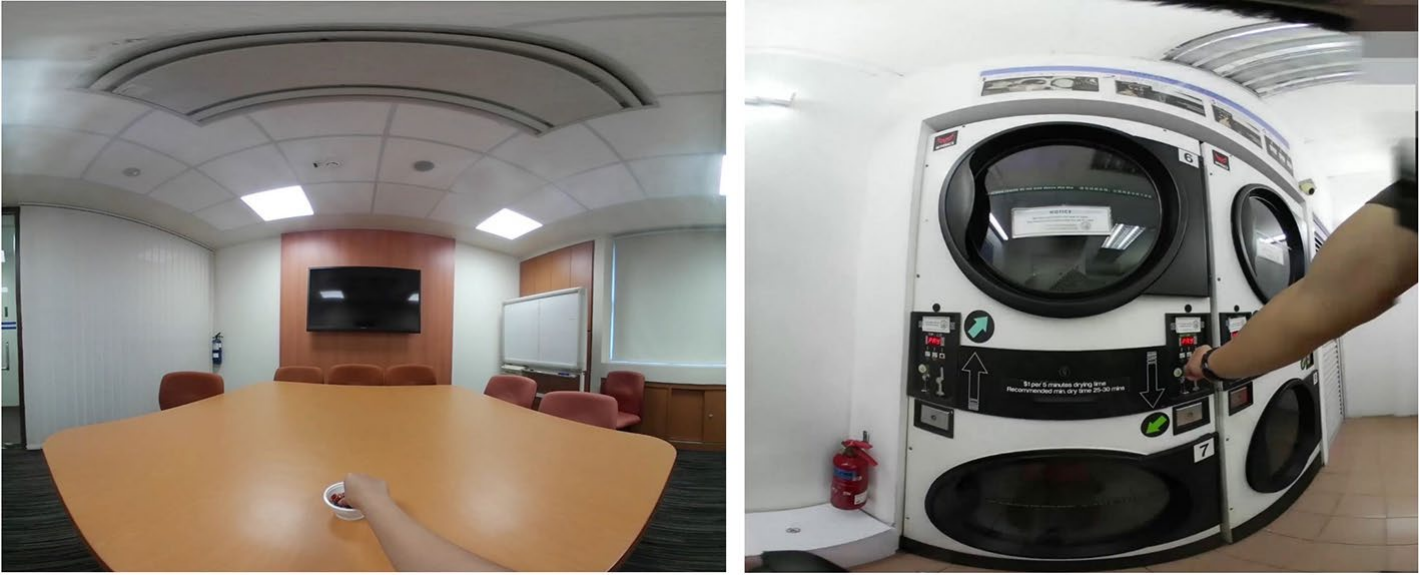
Screenshots depicting first-person 360-degree videos of an M&M being consumed (left) and a coin being inserted into a laundry machine (right).

The stimulus 360-degree video, filmed with a *Samsung Gear 360* camera, depicted a person picking up an M&M chocolate from a bowl placed in front of them and consuming it. The control video showed a person picking up a coin from their pocket and slotting it into a laundry machine. Both male and female versions of the stimulus and control videos were recorded, and the actors’ faces edited out in post-processing. Screenshots of the videos used are shown in Fig. 1.

Participants were randomly assigned one of the three conditions, where they watched the 360-degree video through an *HTC Vive Pro* head mounted display (HMD). Participants were shown videos that corresponded with the gender they identified with. After watching the sequence of 360-degree videos they were assigned to, participants were presented with a bowl containing 40 g of M&Ms and asked to help with a taste test. Participants were instructed to consume the food item and rate it on a few dimensions. They were told that they could eat as little or as much as they needed in order to make good ratings. This technique to measure food consumption was validated in past studies^1,47^. Once participants had completed the taste test, they were debriefed and received their participation incentive. The research assistant proceeded to weigh the remaining M&Ms on a digital weighing scale and noted the weight consumed.

#### Results

Results of a one-way analysis of variance (ANOVA) showed that repeatedly watching a 360-degree video of M&Ms being consumed influenced the amount of M&Ms consumed by participants (*F*(2, 39) = 6.38, *p* = 0.004, partial *η*^2^ = 0.25). Tukey pairwise comparisons revealed that participants in the 30-repetition condition (*M* = 2.67, *SD* = 1.05) consumed significantly fewer M&Ms than those in the three-repetition condition (M = 3.98, *SD* = 1.43, 95% CI [− 2.39, − 0.22], *p* = 0.015) and the control condition (*M* = 4.11, *SD* = 1.01, 95% CI [− 2.52, − 0.36], *p* = 0.007). The weight of M&Ms consumed by participants in the three-repetition and control conditions did not differ significantly (95% CI [− 0.95, 1.22], *p* = 0.95).

#### Discussion

Study One tested whether the effects of habituation might exist in a 360-degree consumption video. Our results showed that repeatedly watching a 360-degree video of M&Ms being consumed led to participants consuming fewer M&Ms, suggesting that participants had become habituated to the repeated watching of the food being consumed and perhaps became satiated as well. The lack of significant difference between the three-repetition and control conditions further suggests that simply repeatedly watching 360-degree videos in itself did not prompt habituation, but it was rather the repeated watching of M&Ms being consumed that led to decreased consumption. This supports the idea that habituation is stimulus specific^8,48^.

### Study Two

To further confirm that our findings were due to the proposed habituation effects and were specific to one’s repeated watching of the M&Ms being consumed, a 2 (behavior: eating M&M vs. inserting coin) × 2 (repetitions: 3 vs. 30) between-subjects experiment was conducted. Based on the effect size of 0.25 in Study One, power analyses showed a target sample size of 128. A total of 131 students were recruited but 17 students dropped out before participating, resulting in a final sample of 114 participants. The sample comprised 54 males (47.4%). Their ages (*M* = 22.6, *SD* = 1.48) ranged from 21 to 28.

#### Method

The experimental conditions employed the same 360-degree videos used in Study 1. Four conditions were created in total: three-repetitions of watching M&Ms being consumed (*n* = 28), 30-repetitions of watching M&Ms being consumed (*n* = 29), three-repetitions of watching a coin being inserted into a laundry machine (*n* = 27) and 30-repetitions of watching a coin being inserted (*n* = 30). The procedure was modelled after Study One, with participants similarly tasked with a taste-test after watching the clips in the condition they were assigned to.

#### Results

Results of a two-way ANOVA showed a significant interaction between behavior and repetition (*F*(1, 110) = 10.72, *p* = 0.001, partial *η*^2^ = 0.09). Pairwise comparisons revealed that among participants exposed to watching 360-degree videos of M&Ms being consumed, those in the 30-repetition condition (M = 3.28, *SD* = 1.16) consumed significantly fewer M&Ms than those in the three-repetition condition (M = 5.31, *SD* = 1.61, 95% CI [− 2.81, − 1.25], *p* < 0.001). Among participants exposed to watching 360-degree videos of a coin being inserted into a laundry machine, the weight of M&Ms consumed by those in the three-repetition (M = 4.23, *SD* = 1.55) and thirty-repetition (M = 4.02, *SD* = 1.60) conditions did not differ significantly (95% CI [− 0.58, 0.99], *p* = 0.61).

#### Discussion

Study Two further tested if the effects of repeatedly watching a 360-degree consumption video was due specifically to the repeated watching of M&Ms being consumed, or simply repeated watching of any video, in our case, the control video of a coin being inserted into a laundry machine. Findings from Study Two suggest that habituation from repeatedly watching a 360-degree video of M&Ms being consumed led to decreased consumption of M&Ms, and confirmed that the results from Study One were not due to the control video. All in all, our findings from Studies One and Two support the possibility of leveraging immersive 360-degree videos for decreasing consumption behavior and for use in food-related therapy and treatment. Additional implications are further discussed in the “General discussion” section.

### Study Three

Study Three aimed to extend our understanding of the effects of habituation in 360-degree videos by exploring the influence of additional olfactory cues. We sought to explore if there were any potential interaction effects between repeated exposure to food cues and olfactory cues in the context of 360-degree videos on consumption behavior. A 2 (repetition: 3 vs. 30) × 2 (scent: present vs. absent) between-subjects experiment was conducted. Similar to Study Two, the effect size of 0.25 from Study One was used in power calculations, showing a target sample size of 128. Due to the number of dropouts experienced with Study Two, we increased the number of initial signups for Study Three. We recruited 170 students, with nine dropping out before participating. The final sample of 161 participants, comprised 91 male students (56.5%). Participants’ ages (*M* = 22.8, *SD* = 1.67) ranged from 21 to 28. The number of participants in each condition is presented in Table 1.

**Table 1:** Assignment of treatment conditions (N = 161).

	Scent	
	Present	Absent
Three repetitions	37	39
Thirty repetitions	44	41

#### Method

The stimuli comprised of the same 360-degree video used in the previous studies. In the scent present condition, a cotton bud dipped in chocolate scented aromatic oil was affixed to the front of the participant’s HMD after they had put it on. Participants were not able to see the scented cotton bud as they were viewing through the HMD. This was designed based on previous scent manipulation in VR^1^. In the scent absent condition, participants simply viewed the 360-degree clips without a scented cotton bud. Participants were randomly assigned one of the four conditions, and tasked with a taste-test after completing the viewing.

#### Results

Results of a two-way ANOVA revealed significant main effects for repetition (*F*(1, 157) = 4.20, *p* = 0.04, partial *η*^2^ = 0.03), and for scent (*F*(1, 157) = 5.04, *p* = 0.03, partial *η*^2^ = 0.03). Participants in the 30-repetition condition (*M* = 5.08, *SD* = 1.66) consumed significantly fewer M&Ms than those in the three-repetition condition (*M* = 5.67, *SD* = 1.91), whereas participants in the scent present condition (*M* = 5.05, *SD* = 1.61) consumed significantly fewer M&Ms than those in the scent absent condition (*M* = 5.68, *SD* = 1.94). No significant interaction was detected between repetitions and scent (*F*(1, 157) = 1.27, *p* = 0.26).

#### Discussion

Study Three explored the effects of olfactory cues in a 360-degree video on consumption behavior, and sought to explore interaction effects between olfactory cues and repeated watching of M&Ms being consumed. Our results showed that while repeated exposure and olfactory cues had negative effects on the amount of M&Ms consumed, there were no interaction effects between the two. Hence, while the addition of scent as an olfactory cue in 360-degree videos can lead to decreased consumption behavior, it remains unclear how prolonged exposure to this scent may further influence the rate or intensity of habituation.

## General discussion

In summary, this paper sought to explore if repeatedly watching 360-degree food consumption videos could habituate participants and influence subsequent food consumption. The presence of scent as an olfactory cue was further explored, to examine any potential additive influence. Our findings provided support that participants who repeatedly watched 360-degree videos became habituated more than those who saw fewer repetitions, consuming fewer amount of M&Ms. It was further determined that this habituative influence was specific to the food stimulus, as the difference in consumption behavior was only observed in conditions where participants watched 360-degree videos of consuming the M&M, and not in the control conditions.

Our findings corroborate past studies that support the influence of habituation in other contexts, such as via repeated imagining^6,11^, or within VR environments^28,29^. While habituated responses to food have been determined through repeated imagining or exposure to real-life food cues^6,7^, our findings show that habituation can occur via repeated watching of consumption of food in 360-degree videos. Such immersive virtual environments may better facilitate habituation as compared to repeated imagining^21^, as well as overcome the practical constraints associated with repeated exposure to real-life food cues^10^.

This study also highlights the ability of non-interactive 360-degree videos to influence consumption behavior. One of the criticisms of 360-degree videos is that they are a passive viewing experience and not a fully embodied interaction as compared to VR experiences, and therefore may suffer from a relative lack of immersion^49,50^. This is because in VR experiences, the user controls the character and therefore embodies them. Conversely, as 360-degree videos are based on video recordings, participants may feel they are merely watching the actions of someone else and not living out the experience. Our results suggest that 360-degree videos may be able to create an equally powerful immersive experience. Individuals may become habituated even if they are not explicitly asked to “personalize” the experience such as by imagining themselves enacting a behavior^6^ or by engaging in self-embodied perspective taking in virtual environments^51^.

Next, our findings provide evidence that exposure to an olfactory cue during stimulus-specific watching of 360-degree videos can lead to decreased consumption behavior. This possibly lends further support to embodied cognition, suggesting that the exposure to olfactory food cues alongside visual cues leads to a sensory simulation of tasting the food^1^. This makes sense given that the experience of consuming a food typically involves various senses including sight and smell. There has been some debate as to whether the inclusion of multi-sensory food cues in digital food experiences such as touch or scent will result in increased or decreased consumption as a result of embodied cognition^1,52^. Our findings showed that the exposure to olfactory cues can decrease consumption behavior when viewing 360-degree videos, and that such videos can approximate real-life responses.

Interestingly however, no interaction effect was detected between repeatedly watching a 360-degree food consumption video and the presence of olfactory cues. One reason for this might be that the mere presence of olfactory cues has an additive influence on consumption, but the duration of exposure to smell does not matter. This would challenge literature demonstrating the effects of prolonged exposure to a scent^38^, suggesting that there may be differences in how individuals respond to prolonged exposure to smells in real life and in virtual environments. This finding instead supports one study that shows that exposure time does not have an effect^53^. Another more possible reason could be that there were individual differences in inhibitory control, leading to differences in how the presence of olfactory cues influenced the rate of habituation^54,55^. One final reason is that habituation may occur within a modality more so than across modalities^14^, hence there were no interactive effects when both scent and repeated exposure were present. All in all, more research needs to be done to understand the effects of olfactory cues on repeated exposure to consuming food. It remains unclear how exactly the different sensory activations may function together when individuals are exposed to it over different durations.

Theoretically, this study advances our understanding of habituation via repeated imagining by showing that habituation may similarly occur in immersive media where individuals do not have to repeatedly imagine, but rather can be immersed in repeatedly experiencing the act of consumption. While this study had only looked at 360-degree videos, it may be valuable to compare the effects of habituation and consumption behavior across repeated exposure to consuming food in various immersive media in future. This study also highlights the additive effect of olfactory cues in influencing consumption behavior. The presence of olfactory cues together with immersion in virtual environments might create stronger symbols of individuals’ consumption experience, simulating autonomic responses related to consumption despite not eating the food in real life.

There are also practical implications given the strong interest in using VR technologies for health promotion and in particular in treating eating disorders^56^. First, researchers and health practitioners can consider using 360-degree videos or olfactory cues to supplement existing cue therapy treatments^57^, identifying if there are differences in exposure to food cues and exposure to consumption of food in immersive media. This extends the current purview of VR cue-exposure therapy. Second, as marketers move towards more innovative forms of marketing, leveraging different media and senses, they should be wary of the possibility that their efforts may backfire. If the goal of marketing is to increase motivation to consume or obtain a particular food, it is necessary to consider how long olfactory cues should be present for to achieve the desired effect.

### Limitations and future directions

One major limitation of this paper was that only actual food consumption was measured. While the difference in consumption behavior of M&Ms shows strong support for habituation effects, it remains unknown if this is due to participants’ sensory-specific satiety, which is related to a decreased liking for the M&M, or merely a decreased motivation to obtain the M&M. Some studies have thus attempted to differentiate satiation and habituation effects^18,58^, and future research should therefore explore other measurements of habituation and satiation to identify if there are differences between the two. For example, studies may get participants to rate their hedonic liking towards the presented food stimuli and other food stimuli—satiation should result in a decreased liking towards all presented food stimuli after repeated exposure to one specific stimuli, whereas habituation is stimulus-specific and should only relate to the food presented. This has implications for binge-eating behaviors, and the longer-term consequences of addiction to overeating in general, compared to addiction to a particular food. Other factors such as salivation upon exposure to the video^59^ can also be measured. Altogether, understanding the specific mechanisms behind decreased consumption can create a more nuanced understanding of the effects of repeated exposures to food cues or consumption of a food, allowing us to better understand its effects in an immersive virtual environment.

Furthermore, while this paper established that watching 360-degree videos of food consumption can induce habituation, only one type of food—chocolate candy, was explored. However, research shows that individuals may have varying perceptions of foods depending on their characteristics^60^. Future studies could therefore explore if the habituation effect manifests in foods of other types, perhaps differentiated along the spectrum of healthiness ranging from fast foods and sodas on one end and salads and fruits on the other. It may also be worthwhile to explore other types of olfactory cues, since the use of a chocolate scent was an aromatic scent, while other unappetizing scents may have distinct effects^4^.

Next, because all three of our studies used a sample of university students, we are unable to generalize these findings to those with eating disorders. For example, a study by Geisel et al.^22^ found that repeated imagining of alcohol consumption only decreased consumption for healthy individuals, but not for alcohol-dependent patients. Future studies should therefore explore the effects of habituation and satiation in 360-degree videos amongst more specific populations.

Finally, future studies should also consider the longer-term effects of habituation and embodied cognition, perhaps comparing between the two. Most studies on habituation merely look at consumption behavior immediately after the experimental exposure to the food item^11,18,61^, and only some have explored the lasting effects of habituation^62^. It is also widely accepted that the effects of habituation will disappear over time^8^. Hence, future studies should explore the longer-term effects of habituation to identify if there are optimal amounts of repeated exposures or an optimal duration between exposures to decrease consumption behavior. If this were the case, 360-degree consumption experiences could have an even stronger role to play in therapy and treatment for eating disorders.

## Data Availability

The data generated and analyzed for the study is available from the corresponding author on reasonable request.
